# Antimicrobial Resistance and Virulence Factors of *Proteus mirabilis* Isolated from Dog with Chronic Otitis Externa

**DOI:** 10.3390/pathogens11101215

**Published:** 2022-10-21

**Authors:** Jun Kwon, Myoung-Hwan Yang, Hyoung-Joon Ko, Sang-Guen Kim, Chul Park, Se-Chang Park

**Affiliations:** 1 Laboratory of Aquatic Biomedicine, College of Veterinary Medicine and Research Institute for Veterinary Science, Seoul National University, Seoul 08826, Korea; 2 Department of Veterinary Internal Medicine, Jeonbuk National University, Iksan 54596, Korea

**Keywords:** otitis externa, antibiotic resistance, *Proteus mirabilis*, multidrug resistance

## Abstract

Otitis externa is among the most prevalent diseases in dogs. If the underlying cause is not addressed, bacterial reinfection becomes frequent, necessitating antibiotic administration for an extended period of time. Prolonged treatment promotes the emergence of antibiotic-resistant bacteria and increases the risk of their transmission from animals to humans. This study aimed to analyze the antibiotic resistance pattern of the emerging pathogen *Proteus mirabilis* to identify bacterial virulence and antibiotic selection. Samples were collected from randomly encountered dogs with chronic otitis externa. Thirty-two strains of *P. mirabilis* were isolated and identified, using MALDI-TOF. The Kirby-Bauer disk diffusion method was used to assess the antibiotic susceptibility of *P. mirabilis* to 11 antibiotics. The isolates (n = 32) were most resistant to cefazolin (75%), trimethoprim–sulfamethoxazole (72%), chloramphenicol (72%), amoxicillin–clavulanate (63%), ampicillin (59%), cefepime (56%), ciprofloxacin (53%), aztreonam (50%), ceftazidime avibactam (50%), gentamicin (22%), and amikacin (16%). Moreover, 75% of isolates were found to be multidrug-resistant bacteria. *P. mirabilis* was found to have a high resistance-pattern ratio. Although the exact cause is unknown, continuous antibiotic use is thought to be a major factor. We concluded that antibiotic use must be prudent and selective to prevent antibiotic resistance.

## 1. Introduction

Canine otitis externa is a highly prevalent multifactorial skin disease, accounting for up to 20% of small-animal counseling cases [1]. It is characterized by increased fluid retention, trauma, and obstruction in the pinna and external auditory canal structures. The factors causing otitis externa can be divided into three categories: primary, perpetuating, and predisposing [1,2,3,4,5,6]. Primary factors include parasites, foreign substances, allergies, keratinization disorders, etc. Perpetuating factors exacerbate otitis externa developed by primary factors. They mainly include bacterial and yeast infections [6,7,8]. Predisposing factors are causes that increase the risk of prolonging or developing otitis externa, not causing it directly. Predisposing factors include anatomic and conformational problems, excessive moisture, iatrogenic factors, and obstructive ear diseases [6,7,8].

Otitis externa relapse is frequent if both primary and secondary factors are not concurrently addressed. More than 60% of cases of otitis externa relapse or progress to chronic, with infection accompanying the majority of cases [9]. Persistent infections necessitate the use of massive amounts of antibiotics, promoting the emergence of antimicrobial-resistant bacteria (AMR) [10,11]. There are numerous factors that contribute to the emergence of AMR, but the most important factor is antimicrobial use (AMU), which promotes the selection of resistant bacteria [10,11,12,13,14,15,16].

AMR bacteria can be transmitted from animals to humans through direct or indirect contact with contaminated food, surroundings, and the environment [17,18,19]. Dogs and cats have been considered to be both reservoirs and transmission vectors [17,18,20,21]. Multiple studies have described the possibility of AMR transmission from dogs and cats (companion animals) to humans [22,23,24,25]. Moreover, the population of companion animals is continuously growing (an increase of approximately 2 million between 2000 and 2020, according to a PFMA survey) [26], thereby increasing the likelihood of bacterial zoonotic diseases.

*Proteus mirabilis*, a Gram-negative bacterium, is an emerging pathogen in veterinary and human medicine [27]. *P. mirabilis* is found in diverse habitats, including soil and animal urinary and digestive tracts, with the most common infection site being the upper urinary tract [28,29,30,31,32,33,34,35]. *P. mirabilis* infection in the upper urinary tract can cause urolithiasis, permanent kidney damage, bacteremia, and sepsis [36,37,38,39,40]. Bacteremia and sepsis induced by *P. mirabilis* were associated with a higher mortality rate than those caused by other pathogens [39,40,41,42]. Moreover, because multidrug-resistant bacteria have been reported, the clinical implications of these bacteria on public health are crucial.

In this study, the antibiotic resistance pattern of *P. mirabilis* isolated from the ears of dogs with chronic otitis externa that had been treated with antibiotics for more than a year was determined. This study aimed to assess the antibiotic resistance of *P. mirabilis* caused by continuous antibiotic use and to encourage the prudent use of antibiotics.

## 2. Materials and Methods

### 2.1. Sample Collection and Identification of Proteus mirabilis Isolates

*Proteus mirabilis* strains were isolated from sixty external ear-canal swab samples procured from two individual animal hospitals (Seoul and Gyeongsangnam-do). The Amies transport medium (YUHAN LAB TECH Co., Ltd., Seoul, Korea) was used for the swab sampling. Samples were collected for two years from canines with chronic otitis externa (over one year of treatment). The swabs were placed in Eppendorf tubes with phosphate-buffered saline solution and vortexed vigorously. The supernatants were spread on Columbia blood agar (5% sheep blood; Oxoid, Hampshire, UK) and incubated overnight at 37 °C. Suspected *P. mirabilis* colonies were chosen by colony morphology and sub-cultured onto fresh tryptic soy agar (TSA; Difco, Detroit, MI, USA) and incubated overnight at 37 °C; this process was repeated three times. Following the isolation of pure colonies, the isolates were identified by using mass spectrometry. Pure isolated bacterial proteins were extracted for identification using matrix-assisted laser desorption/ionization/time-of-flight mass spectrometry (MALDI Biotyper; Bruker Daltonics, Bremen, Germany) in accordance with the ethanol/formic acid protocol [43]. The obtained spectra were compared to the patented manufacturer’s library. Bacteria were identified based on similarity log scores according to the standard Bruker interpretative criteria: a score ≥2.0 was accepted for species assignment, and a score ≥1.7 and ≤2.0 was accepted for genus identification. Bacteria were stored at −70 °C, in tryptic soy broth (TSB; Difco, Detroit, MI, USA) containing 15% glycerol.

### 2.2. Antimicrobial Susceptibility Test

The Kirby-Bauer disk diffusion method, as described in the Clinical and Laboratory Standards Institute (CLSI) [44], was conducted to examine the susceptibility of bacterial isolates to 11 commonly used antibiotics (7 classes), namely ampicillin (penicillin, AMP; 10 µg), amoxicillin–clavulanate (β-lactam inhibitors, AMC; 20/10 µg), ceftazidime avibactam (β-lactam inhibitors, CZA; 30/20 µg), gentamycin (aminoglycoside, CN; 10 µg), cefazolin (cephem, KZ; 30 µg), cefepime (cephem, FEP; 30 µg), aztreonam (monobactam, ATM; 30 µg), amikacin (aminoglycoside, AK; 30 µg), trimethoprim–sulfamethoxazole (folate pathway antagonist, SXT; 1.25/23.75 µg), ciprofloxacin (quinolone, CIP; 5 µg), and chloramphenicol (C; 30 µg). The investigated data were listed and formatted as heatmap plots by using Microsoft Excel 2020. *P. mirabilis* isolates resistant to at least three classes of antimicrobials were categorized as MDR isolates [45]. The multiple antibiotic resistance (MAR) index values were calculated as described in Reference [46].

### 2.3. Phenotypic Detection of Extended Spectrum Beta-Lactamase (ESBL)

To detect ESBL, a double-disk synergy test was performed, as described in the CLSI guidelines [44]. Briefly, McFarland bacterial suspensions (0.5 mL) were spread on Mueller–Hinton agar (Difco, USA). Ceftazidime (30 µg), cefotaxime (30 µg), ceftazidime/clavulanic acid (30/10 µg), and cefotaxime/clavulanic acid (30/10 µg) discs were placed on agar plates. The plates were then incubated at 37 °C for 18 h. The diameters of the inhibition zones were measured.

### 2.4. Genotypic Detection of Antibiotic Resistance, Virulence, and Highly Conserved Genes

To investigate virulence and antimicrobial-resistance genes, conventional PCR was performed to amplify 20 antimicrobial-resistance genes [47] and 5 virulence genes (*ureC*, *rabA*, *zapA*, *hpmA*, and *hlyA*) [48]. The genes encoding β-lactam (*bla_CTX-M_*, *bla_OXA_*, and *bla_SHV_*), aminoglycoside (*aaC1*, *aaC2*, *aaC3*, and *aac(6′)-lb-cr*), tetracycline (*tetA* and *tetB*), sulfonamide (*sul1*, *sul2*, and *sul3*), quinolone (*qnrA*, *qnrB*, *qnrC*, and *qnrS*), macrolide (*mefA* and *mrsD*), and phenicol (*stcM* and *cmlA*) resistance proteins were subjected to PCR. The target PCR products were gel-extracted and sequenced for confirmation. The investigated data were listed and formatted as heatmap plots by using Microsoft Excel 2020.

A conserved gene (*atpD*) was also sequenced for phylogenetic investigation [48].

### 2.5. Phylogenic Investigation of P. mirabilis Isolates

For phylogenetic analysis, the genome sequences of the conserved gene *atpD* were aligned with the sequences obtained from the GenBank^®^ database, using ClustalW [49]. Phylogenetic trees were constructed in MEGA v10.1.8, using the maximum likelihood method with 1000 bootstrap replications [50].

## 3. Results

### 3.1. Antimicrobial Susceptibility Profile

The antibiotic susceptibility test results categorize isolates into three groups in terms of number and ratios: susceptible, intermediate, and resistant (Table 1). The majority of isolates was resistant to cefazolin (75%, 24/32), trimethoprim–sulfamethoxazole (72%, 23/32), and chloramphenicol (72%, 23/32). Ampicillin (59%, 19/32) and amoxicillin–clavulanate (63%, 20/32) follow the chloramphenicol and trimethoprim–sulfamethoxazole in terms of resistance ratio. Almost half of the isolates (56%, 18/32) were resistant to fourth-generation cephalosporin and cefepime, and 6% (2/32) were intermediate resistant strains. Amikacin was shown to be the most susceptible antibiotic to the clinical isolates.

The MAR index is presented in Supplementary Table S1. Twenty isolates were over 0.5 MAR index, and the mean value of the MAR index was 0.534. Among them, two strains were 1 MAR index, and six strains were 0 in MAR index.

**Figure 1:**
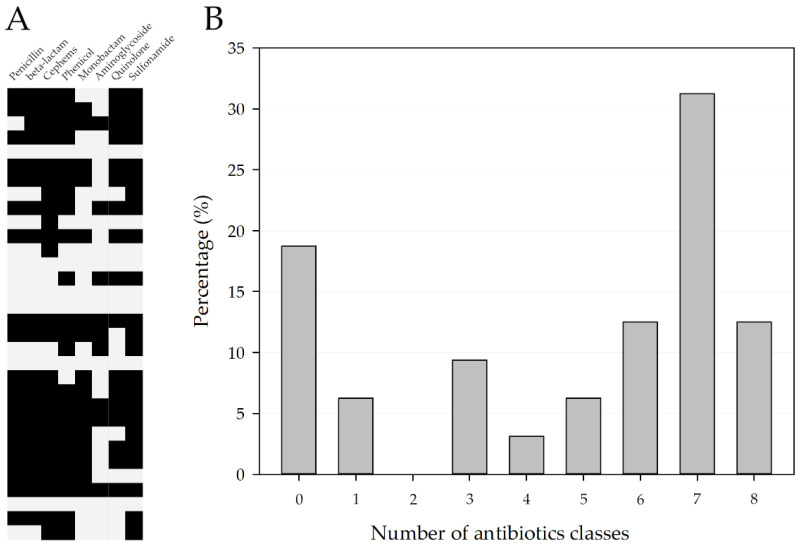
(**A**) Heatmap of antibiotics class resistance. Black, resistance. (**B**) Percentage of *P. mirabilis* strains in accordance with the number of resistance antibiotic classes.

**Figure 2:**
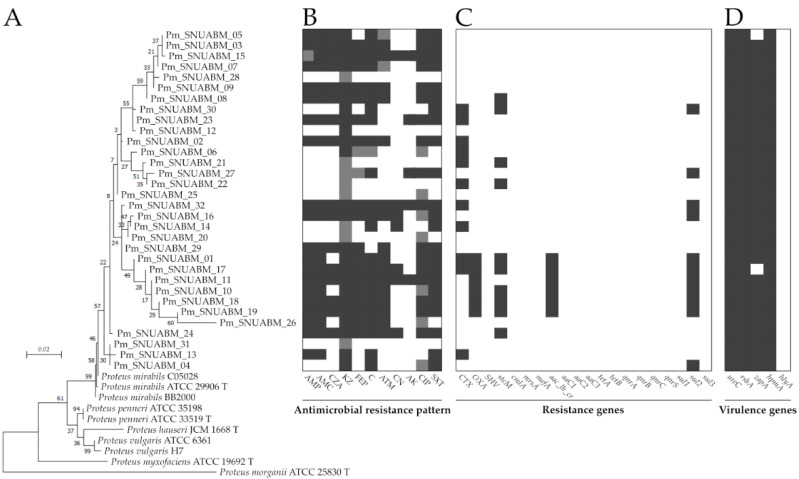
(**A**) Comparative analysis of *P. mirabilis*, using *atpD* gene sequences. (**B**) Antimicrobial-resistance pattern of *P. mirabilis* strains isolated from chronic otitis externa in dogs. (**C**,**D**) Heatmap of antibiotic resistance and virulence genes. Type strains are designated with a “T”.

The antimicrobial-resistance patterns of *the P. mirabilis* isolates are presented in Figure 1 and Figure 2B. Most isolates (81.25%, 26/32) were resistant to at least one antibiotic. Three isolates were resistant to all antibiotics used in this study. Additionally, 75% (24/32) of the isolates were found to be MDR.

In the ESBL test, eight isolates were found to be resistant to ceftazidime (≤22 mm) and cefotaxime (≤27 mm), indicating that the isolates may produce ESBL. Among them, four isolates demonstrated a ≥5 mm increase in the zone diameter compared to antimicrobial agents in combination with clavulanic acid, confirming the presence of ESBL-producing strains. 

### 3.2. Genotypic Description of Clinical Isolates of P. mirabilis

Resistance genes were detected by using conventional PCR (Figure 2C). Among these genes, many of them (*bla_SHV_*, *tetA*, *tetB*, *cmlA*, *mefA*, *mrsD*, *aaC1*, *aaC2*, *aaC3*, *sul1*, *sul3*, *qnrA*, *qnrB*, *qnrC*, and *qnrS*) were not detected. The β-lactamase genes, *bla_CTX-M_* and *bla_OXA_*, were detected in 11 (34%) and 6 (19%) isolates, respectively. The phenicol efflux gene, *stcM*, was found in 10 (31%) isolates. The gene *sul2*, a sulfonamide-resistance gene, was detected in 11 (34%) isolates. The aminoglycoside efflux gene, *aac(6′)-lb-cr*, was detected in 6 (19%) isolates.

Virulence factors showed high prevalence, except *for hlyA* (Figure 2D). The prevalence of *ureC* (32; 100%), *rsbA* (32; 100%), *hmpA* (32; 100%), and *zapA* (30; 94%) was determined (Figure 2D). The *hlyA* gene was not detected (0; 0%) (Figure 2D).

The phylogeny demonstrated that the isolated strains were clustered with *P. mirabilis* strains sequences obtained from GeneBank database (Figure 2A). 

## 4. Discussion

Recent studies have focused on the emergence of antibiotic-resistant *P. mirabilis* [27,29]. This study aimed to identify the ratio of antibiotic-resistance patterns of *P. mirabilis* clinical isolates through antibiotic resistance evaluation and to aid in the selection of susceptible antibiotics. Numerous antimicrobial-resistant strains of the widespread bacterium *P. mirabilis* have been identified. Although it is mainly associated with urinary tract infections, it is also the third most common cause of otitis externa in dogs [51,52]. Persistent reinfection is common in chronic otitis externa in dogs, and it is often accompanied by recurrent antibiotic doses. Known to some extent, AMR bacteria are selected by antimicrobial usage. Repeated antibiotic use leads to an increase in the incidence of antibiotic resistant bacteria. It is particularly common with sublethal doses of antibiotics [53]. 

As indicated in the results, the clinical isolates were resistant to the most frequently used or first-line antibiotics prescribed for otitis externa infection. TE is one of the most frequently used antibiotics in the topical treatment of ear infections [52]. The penicillin class of antibiotics, such as ampicillin and amoxicillin–clavulanic acid, is also often used in clinical settings [53,54]. Relatively high resistance to ampicillin and amoxicillin–clavulanate was observed in this study. On the other hand, amikacin was a susceptible antibiotic to most of the isolates. There were isolates (6%, 2/32), which were only susceptible to amikacin. This is presumably because the antibiotics are not used as per oral (PO) agent. Meaning, it is not easy and preferred to maintain drug administration in household condition. Moreover, the nephrotoxicity of amikacin is one of the reasons why the drug is not frequently used [55]. 

Various discrepancies in antimicrobial-resistance phenotype and genotype were observed. Upon phenotypic detection of antibiotic resistance, all of the isolates exhibited overall antibiotic resistance. On the other hand, ampicillin and amoxicillin–clavulanate resistances were observed in about 60% of the strains. However, the β-lactamase genes bla*_CTX_*and bla*_OXA_* were detected only in 34% (11/32) and 18% (6/32) of isolates, respectively. Furthermore, the tetracycline-resistance genes, *tetA* and *tetB*, were not detected. Other resistance genes were detected at a low frequency compared to the phenotypic resistance ratio. This discrepancy may be due to our list of antimicrobial-resistance gene targets [56]. The lack of detection of antimicrobial-resistance genes does not mean confirmation of antibiotic susceptibility [56]. We believe that the other antimicrobial-resistance genes that our target primers did not detect are present in the bacterial genome [56]. 

The virulence factors (*ureC*, *rsbA*, *zapA*, *hpmA*, and *hlyA*) were detected in high prevalence in the isolates. These genes are related to swarming modulation (*rsbA*), urease enzyme production (*ureC*), IgA protease enzyme production (*zapA*), and cytotoxic hemolysin (*hpmA*) [48,57]. Because the *P. mirabilis* infections are dependent on various virulence factors [58], the high prevalence of virulence factors indicates the possibility of transmission from dogs to humans when they share living space. The *ureC* gene demonstrates that the bacteria can metabolize urea, which is distributed in serum. Urea metabolism can disturb local pH and tissue damage [59]. Furthermore, a disturbance of the pH can have an effect on antibiotic effectiveness [60,61]. The existence of the *rsbA* gene suggests that the bacteria are able to detect signals and respond in order to prolong their survival by swarming or internalization [61]. Moreover, *zapA* encodes metalloprotease, which can cleave various peptides and proteins. This enzyme degrades immunoglobulin A and antimicrobial peptides [62,63]. Therefore, the bacteria can defend themselves from and survive host responses [63]. Furthermore, enzyme ZapA would contribute to nitrogen and carbon acquisition by metabolizing host proteins [63]. Moreover, *hpmA* is a hemolysin gene which contributes to pathogenicity [57]. HpmA hemolysin demonstrates cytotoxic activity not only to red blood cells, but also to several type of cells, such as human bladder epithelial cells, monocytes, and B-cell lymphomal cells [57].

As antibiotics emerged and ushered in a medical revolution, they became an indispensable part of medical care. However, the widespread use of antibiotics has led to the rise of antibiotic-resistant bacteria. Antibiotic resistance has emerged as one of the greatest public health threats. Therefore, it is necessary to develop antibiotic alternatives, such as bacteriophages, bacteriocin, and antimicrobial peptides [58,64,65]. This study supports the severity of antimicrobial-resistance emergence and the need for the development of antibiotic alternatives.

## 5. Conclusions

*P. mirabilis*, which is primarily associated with urinary tract infections, is an emerging pathogen due to increased antimicrobial resistance. Furthermore, it is known to be one of the major infectious agents of canine otitis externa. The results demonstrated a high phenotypic antibiotic resistance in *P. mirabilis* to multiple classes of antibiotics, necessitating the need for prudent antibiotic use to prevent the acquisition of antibiotic resistance in *P. mirabilis*. Further research is required to develop antibiotic alternatives against MDR bacteria.

## Figures and Tables

**Table 1:** Antimicrobial-susceptibility profile of *Proteus mirabilis* (N = 32) isolated from dogs with chronic otitis externa.

Antibiotics	Susceptible	Intermediate	Resistant
AMP	12 (38%)	1 (3%)	19 (59%)
AMC	12 (37%)		20 (63%)
CN	25 (78%)		7 (22%)
ATM	14 (44%)	2 (6%)	16 (50%)
KZ		8 (25%)	24 (75%)
AK	27 (84%)		5 (16%)
CIP	8 (25%)	7 (22%)	17 (53%)
SXT	9 (28%)		23 (72%)
C	8 (25%)	1 (3%)	23 (72%)
CZA	16 (50%)		16 (50%)
FEP	12 (38%)	2 (6%)	18 (56%)

AMP, ampicillin; AMC, amoxicillin–clavulanic acid; CN, cephazolin; ATM, aztreonam; KZ, gentamicin; AK, amikacin; CIP, ciprofloxacin; SXT, trimethoprim sulfamethoxazole; C, chloramphenicol; CZA, ceftazidime; FEP, cefepime.

## Data Availability

Not applicable.

## Supplementary Materials

The following supporting information can be downloaded at: https://www.mdpi.com/article/10.3390/pathogens11101215/s1. Table S1: Multiple antibiotic resistance (MAR) index of *Proteus mirabilis* strains isolated from chronic otitis externa in dogs.
