# Supplementary material for: Antimicrobial Resistance and Virulence Factors of Proteus mirabilis Isolated from Dog with Chronic Otitis Externa
Source: Pathogens. 2022 Oct 21;11(10):1215. doi: 10.3390/pathogens11101215 (PMC9612330; doi:10.3390/pathogens11101215)
Supplement: Supplementary file 1 [file pathogens-11-01215-s001.zip › pathogens-1962516-supplementary.pdf]

Supplementary Table S1. Multiple antibiotic resistance (MAR) index of *Proteus mirabilis* strains isolated from chronic otitis externa in dogs. The isolates were listed in order of phylogenetic tree in Figure 2.

***Proteus* isolates**	**MAR index**
Pm_SNUABM_05	0.636
Pm_SNUABM_03	0.818
Pm_SNUABM_15	0.909
Pm_SNUABM_07	0.727
Pm_SNUABM_28	0
Pm_SNUABM_09	0.818
Pm_SNUABM_08	0.818
Pm_SNUABM_30	0.272
Pm_SNUABM_23	0.818
Pm_SNUABM_12	0.090
Pm_SNUABM_02	0.818
Pm_SNUABM_06	0.090
Pm_SNUABM_21	0
Pm_SNUABM_27	0.363
Pm_SNUABM_22	0
Pm_SNUABM_25	0
Pm_SNUABM_32	1
Pm_SNUABM_16	0.818
Pm_SNUABM_14	0.272
Pm_SNUABM_20	0
Pm_SNUABM_29	0.727
Pm_SNUABM_01	0.727
Pm_SNUABM_17	0.909
Pm_SNUABM_11	1
Pm_SNUABM_10	0.636
Pm_SNUABM_18	0.818
Pm_SNUABM_19	0.818
Pm_SNUABM_26	0.545
Pm_SNUABM_24	0.909
Pm_SNUABM_31	0
Pm_SNUABM_13	0.454
Pm_SNUABM_04	0.272
Mean value	0.534
